# Commentary: Tractography-Activation Models Applied to Subcallosal Cingulate Deep Brain Stimulation

**DOI:** 10.3389/fnana.2015.00148

**Published:** 2015-11-19

**Authors:** Ioannis N. Mavridis

**Affiliations:** Department of Neurosurgery, ‘K.A.T.-N.R.C.’ General Hospital of AtticaAthens, Greece

**Keywords:** deep brain stimulation, functional neurosurgery, structural magnetic resonance imaging, subcallosal cingulate white matter, tractography activation models

Structural magnetic resonance imaging (MRI) techniques are a representative example of the current progress in neuroimaging and enable imaging neuroanatomy to be directly applied to clinical practice. Particularly the field of stereotactic and functional neurosurgery has begun to gain remarkable benefits from this revolutionary evolution. Such techniques are ideal for studying clinical populations considered for functional neurosurgery, especially when crucial structures of complicated neuronal networks are targeted. Another benefit is the possibility for a personalized treatment, as was well illustrated in the recent article by Lujan et al. (2013) regarding tractography-activation models applied to subcallosal cingulate (SCC) deep brain stimulation (DBS). The purpose of this communication is to comment on this interesting article.

Lujan et al. (2013) presented tractography activation models (TAMs)-based predictions from SCC DBS in a patient suffering from major depressive disorder. They applied patient-specific TAMs to identify pathways potentially modulated by DBS. The basic components of TAMs included anatomic and diffusion-weighted imaging data, probabilistic tractography, finite element models of the generated electric field and application of this field to multi-compartment cable models of axons to predict action potential generation in specific pathways, (Lujan et al., 2013). Their target's choice was wise in the context of their study's purpose. The connectivity of SCC with the frontal cortex, cingulate cortex, nucleus accumbens, thalamus, amygdala, hippocampus and brainstem, which was depicted in their imaging results (Lujan et al., 2013), shows the importance of such imaging modalities in the manipulation efforts of complex brain networks.

The findings of Lujan et al. (2013) suggested that small differences in electrode location can generate substantial differences in the directly activated pathways and confirmed widespread network changes associated with DBS induced antidepressant effects. Their case suggests that activation of a critical mass of a unique combination of cortical, sub-cortical and cingulate pathways may be necessary for therapeutic benefit (Lujan et al., 2013). Although, I agree with the authors that such efforts are in the right direction to assist in the definition of target pathways for DBS applications (Lujan et al., 2013), validation of TAM predictions remains a modern necessity.

DBS is nowadays an established therapy for selected patients suffering from various neurologic disorders and is also currently used for treating carefully selected patients suffering from some common psychiatric disorders such as treatment-resistant depression (TRD) and obsessive-compulsive disorder (Mavridis, 2012). DBS in psychiatric disorders represents a unique source of information to probe results gained in functional, structural and molecular neuroimaging studies *in vivo* (Höflich et al., 2013). Further, the availability of DBS data in stereotactic space may facilitate the investigation and interpretation of treatment and side effects of DBS by comparing these to neuroimaging results (Höflich et al., 2013).

We currently know that DBS acts not only in the brain area where it is being applied, but chronic stimulation activates axons located in its scope and can exert its effects in distant areas. Considering this, DBS target identification should be based on techniques that identify white matter tracts, such as tractography (Torres et al., 2014), especially when targeting white matter structures such as the SCC. Therefore, tractography has been used in the field of DBS to clarify relevant aspects in the selection of targets and in evaluating its therapeutic effects in movement disorders, psychiatric diseases and pain (Torres et al., 2014). It can help to estimate anatomic regions of DBS-evoked activation (Mädler and Coenen, 2012; Hartmann et al., 2015). The knowledge of this anatomic distribution may contribute in the prediction of efficacy and side effects, and can be used to improve the therapeutic effectiveness of individual adjustments in DBS patients (Hartmann et al., 2015). It provides also opportunities to improve clinical selection of surgical targets and stimulation settings (Lujan et al., 2012).

Furthermore, SCC DBS is an evolving investigational treatment for depression, probably by modulating the activity within a network of brain regions involved in mood regulation (Riva-Posse et al., 2014). Riva-Posse et al. (2014) used diffusion tensor imaging (DTI) of white matter connections within this network to identify those critical for successful antidepressant response in 16 patients with TRD who received SCC DBS. Probabilistic tractography was used to delineate the white matter tracts traveling through each activation volume. All DBS responders shared bilateral pathways from their activation volumes to medial frontal cortex, rostral and dorsal cingulate cortex, and subcortical nuclei (Riva-Posse et al., 2014). Thus, patient-specific activation volume tractography modeling may assist in the identification of critical tracts that mediate SCC DBS antidepressant response (Riva-Posse et al., 2014).

Several studies of DBS for TRD have shown antidepressant effects and recently, DBS to the medial forebrain bundle (a key structure of the reward system) has yielded promising results (Schlaepfer et al., 2014). In this case the target is also selected based on DTI tractography. Even more recently, it was suggested that analysis of transient behavior changes during SCC DBS and subsequent identification of unique connectivity patterns may provide a biomarker of a rapid-onset depression switch to guide surgical implantation and to refine and optimize selection algorithms of contacts in long-term stimulation for TRD (Choi et al., 2015).

Thinking of cautions that unavoidably come with new technologies, we should keep in mind that DTI tracts are complex mathematical objects and the validity of tractography-derived information in clinical settings has yet to be fully established (Pujol et al., 2015). Tractography could fundamentally change our understanding of DBS, but it could also seriously harm patients if we do not recognize and attempt to understand its limitations.

In conclusion, tractography appears as an imaging tool of great value in functional neurosurgery. It can reveal the spectrum of network changes associated with DBS and also the differences in the activated pathways which result from small differences in electrode location. Thanks to such modern structural MRI modalities, as tractography, we nowadays know that activation of a critical mass of unique anatomic connectivity may be necessary for achieving the desired DBS-induced therapeutic benefit. Finally, tractography enables personalized treatment, which appears to be crucial for a successful neuromodulation procedure, especially a DBS application.

## Author contributions

Dr. IM is the sole contributor of this article.

### Conflict of interest statement

The author declares that the research was conducted in the absence of any commercial or financial relationships that could be construed as a potential conflict of interest.

## Abbreviations

DBS: deep brain stimulation
DTI: diffusion tensor imaging
MRI: magnetic resonance imaging
SCC: subcallosal cingulate
TAMs: tractography activation models
TRD: treatment-resistant depression.

## References

[B1] Choi K. S. Riva-Posse P. Gross R. E. Mayberg H. S. (2015). Mapping the “Depression Switch” during intraoperative testing of subcallosal cingulate deep brain stimulation. JAMA Neurol. 72, 1252–1260. 10.1001/jamaneurol.2015.2564 26408865 PMC4834289

[B2] Hartmann C. J. Chaturvedi A. Lujan J. L. (2015). Quantitative analysis of axonal fiber activation evoked by deep brain stimulation via activation density heat maps. Front. Neurosci. 9:28. 10.3389/fnins.2015.00028 25713510 PMC4322637

[B3] Höflich A. Savli M. Comasco E. Moser U. Novak K. Kasper S.. (2013). Neuropsychiatric deep brain stimulation for translational neuroimaging. Neuroimage 79, 30–41. 10.1016/j.neuroimage.2013.04.065 23631986

[B4] Lujan J. L. Chaturvedi A. Choi K. S. Holtzheimer P. E. Gross R. E. Mayberg H. S.. (2013). Tractography-activation models applied to subcallosal cingulate deep brain stimulation. Brain Stimul. 6, 737–739. 10.1016/j.brs.2013.03.008 23602025 PMC3772993

[B5] Lujan J. L. Chaturvedi A. Malone D. A. Rezai A. R. Machado A. G. McIntyre C. C. (2012). Axonal pathways linked to therapeutic and nontherapeutic outcomes during psychiatric deep brain stimulation. Hum. Brain Mapp. 33, 958–968. 10.1002/hbm.21262 21520343 PMC5032841

[B6] Mädler B. Coenen V. A. (2012). Explaining clinical effects of deep brain stimulation through simplified target-specific modeling of the volume of activated tissue. Am. J. Neuroradiol. 33, 1072–1080. 10.3174/ajnr.A2906 22300931 PMC8013266

[B7] Mavridis I. N. (2012). Stereotactic Neurosurgical Anatomy of the Nucleus Accumbens. Ph.D. Thesis, National and Kapodistrian University of Athens (School of Medicine), Athens.

[B8] Pujol S. Wells W. Pierpaoli C. Brun C. Gee J. Cheng G.. (2015). The DTI Challenge: toward standardized evaluation of diffusion tensor imaging tractography for neurosurgery. J. Neuroimaging 25, 875–882. 3 10.1111/jon.12283 26259925 PMC4641305

[B9] Riva-Posse P. Choi K. S. Holtzheimer P. E. McIntyre C. C. Gross R. E. Chaturvedi A.. (2014). Defining critical white matter pathways mediating successful subcallosal cingulate deep brain stimulation for treatment-resistant depression. Biol. Psychiatry 76, 963–969. 10.1016/j.biopsych.2014.03.029 24832866 PMC4487804

[B10] Schlaepfer T. E. Bewernick B. H. Kayser S. Hurlemann R. Coenen V. A. (2014). Deep brain stimulation of the human reward system for major depression–rationale, outcomes and outlook. Neuropsychopharmacology 39, 1303–1314. 10.1038/npp.2014.28 24513970 PMC3988559

[B11] Torres C. V. Manzanares R. Sola R. G. (2014). Integrating diffusion tensor imaging-based tractography into deep brain stimulation surgery: a review of the literature. Stereotact. Funct. Neurosurg. 92, 282–290. 10.1159/000362937 25248076

